# Supplementary material for: Evolving reservoir computers reveal bidirectional coupling between predictive power and emergent dynamics
Source: Patterns (N Y). 2026 Feb 6;7(3):101457. doi: 10.1016/j.patter.2025.101457 (PMC13100675; doi:10.1016/j.patter.2025.101457)
Supplement: Document S1. Figures S1–S7 [file mmc1.pdf]

Patterns, Volume *7*

Supplemental information

Evolving reservoir computers reveal bidirectional coupling between predictive power and emergent dynamics

Hanna M. Tolle, Andrea I. Luppi, Anil K. Seth, and Pedro A.M. Mediano

Supplemental Figures

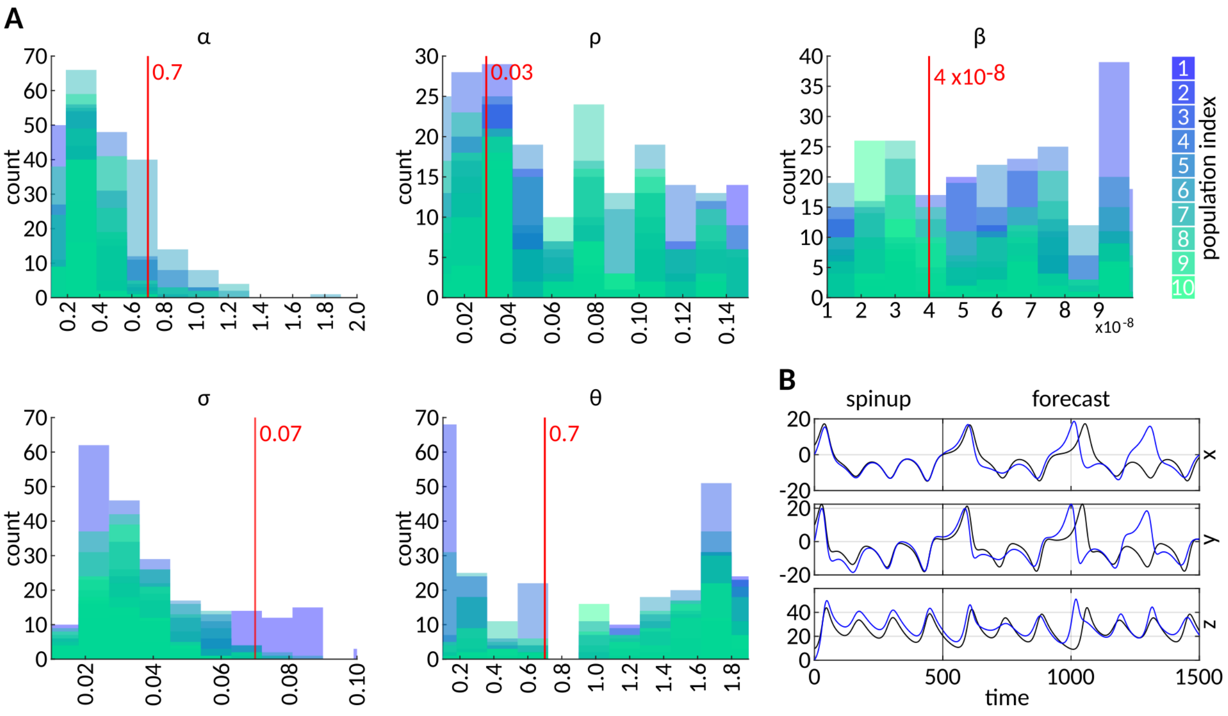

Figure S1. Hyperparameter optimization of bio-inspired reservoir computers for minimal loss.

(**A**) Distributions of hyperparameter values in the final generation of 10 evolved populations. (α: the spectral radius of the reservoir adjacency matrix C; ρ: connection density of C; β: Tikhonov ridge regularization parameter; σ: input strength; θ: input bias) The red lines indicate the hyperparameter values of the best solution across all populations. (**B**) Ground-truth trajectories of each Lorenz variable (black) and RC output (blue), generated by a trained, bio-inspired RC employing the loss-optimal hyperparameters. Black vertical lines demarcate the spinup time from the forecast time. During spinup, the initial 500 time steps of the target environmental trajectory are fed to the reservoir. During forecasting, the RC receives no external input but produces a forecast in a one-step-ahead prediction approach. The readout weights of the RC were computed beforehand on an independently sampled environmental time series for training.

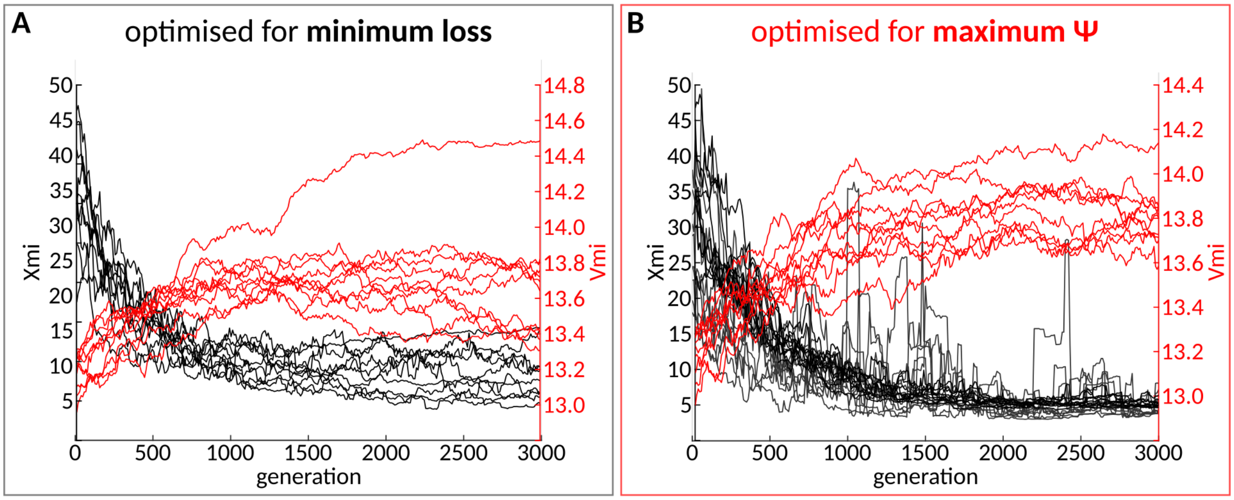

Figure S2. Trajectories of Vmi and Xmi during evolutionary optimization.

(**A**) Average trajectories of $X_{\mathrm{mi}} = \sum_i I(x_{i,t}; y_{t+1})$ (black) and $V_{\mathrm{mi}} = I(y_t; y_{t+1})$ (red) across 10 populations evolved in the Lorenz environment with the objective to minimize prediction loss (*optimization objective I*). (**B**) Same as (A), but for populations evolved with the objective to maximize ψ (*optimization objective II*).

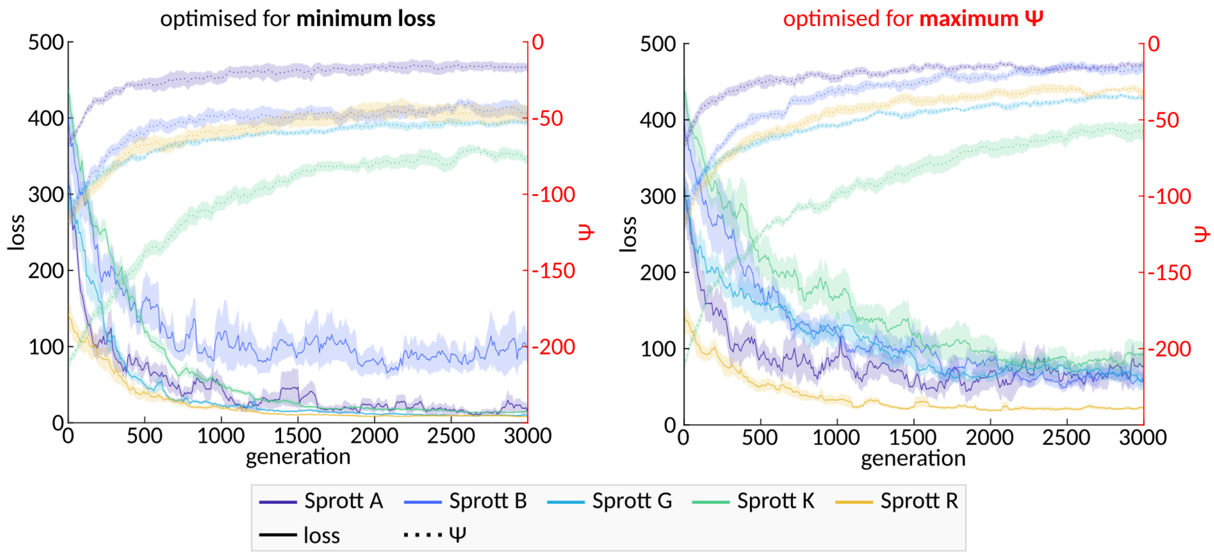

Figure S3. Inverse relationship between loss and ψ during evolutionary optimization of bio-inspired reservoir computers.

Left: Mean trajectories of loss (solid line; left y-axis) and ψ (dotted line; right y-axis), averaged across 10 populations per environment (as indicated by the color and legend), over the course of evolutionary optimization with the objective to minimize loss. Shaded areas indicate standard errors. Right: Analogous to the right plot but for evolutionary optimization with the objective to maximize ψ.

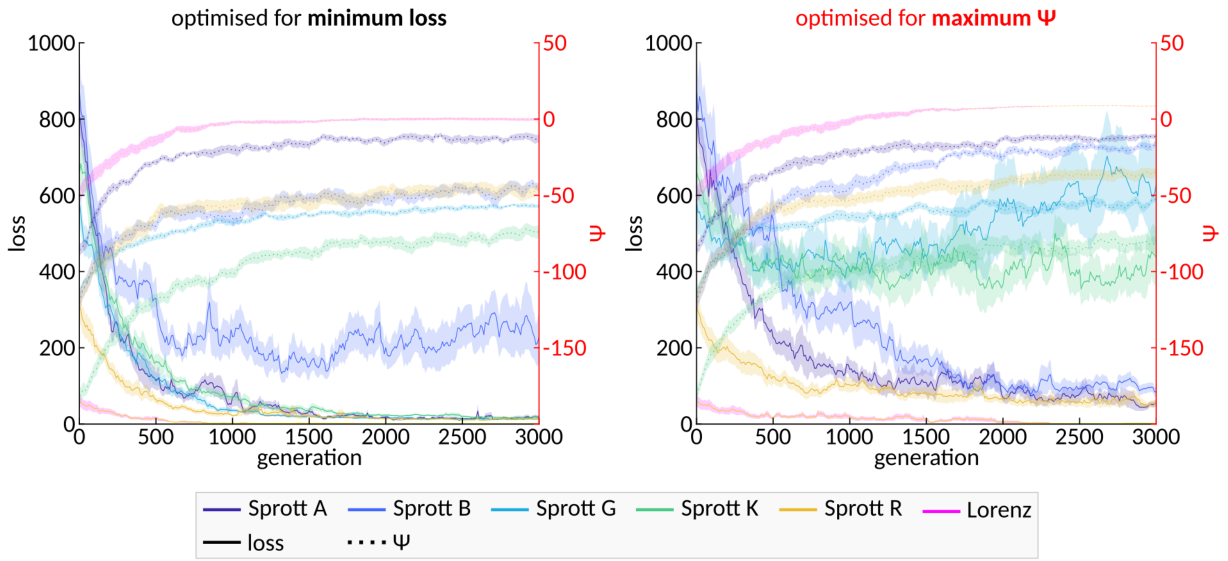

Figure S4. Inverse relationship between loss and ψ during evolutionary optimization of randomly connected reservoir computers.

Left: Mean trajectories of loss (solid line; left y-axis) and ψ (dotted line; right y-axis), averaged across 10 populations per environment (as indicated by the color and legend), over the course of evolutionary optimization with the objective to minimize loss. Shaded areas indicate standard errors. Right: Analogous to the right plot but for evolutionary optimization with the objective to maximize ψ.

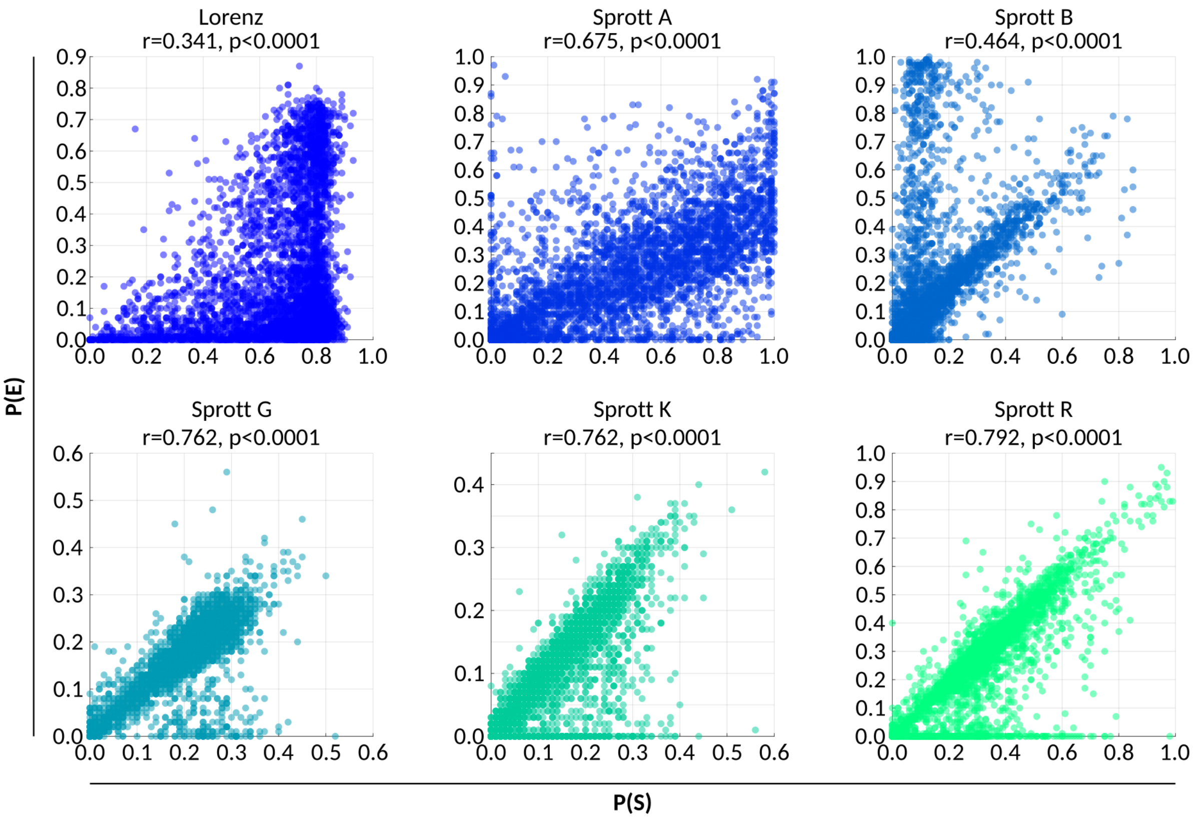

Figure S5. Environment-specific relationships between prediction success and emergence.

Scatter plots of the probability of prediction success $\mathrm{P}(S)$ (loss< 1) against the probability of emergence $\mathrm{P}(E)$ ($\psi > 0$) for each of the six task environments. Each dot represents one of the 4000 RCs with randomly sampled hyperparameters evaluated in Fig. **??**A–C. The plots show that while $\mathrm{P}(S)$ and $\mathrm{P}(E)$ are positively related within all environments, the precise form of this relationship differs across environments.

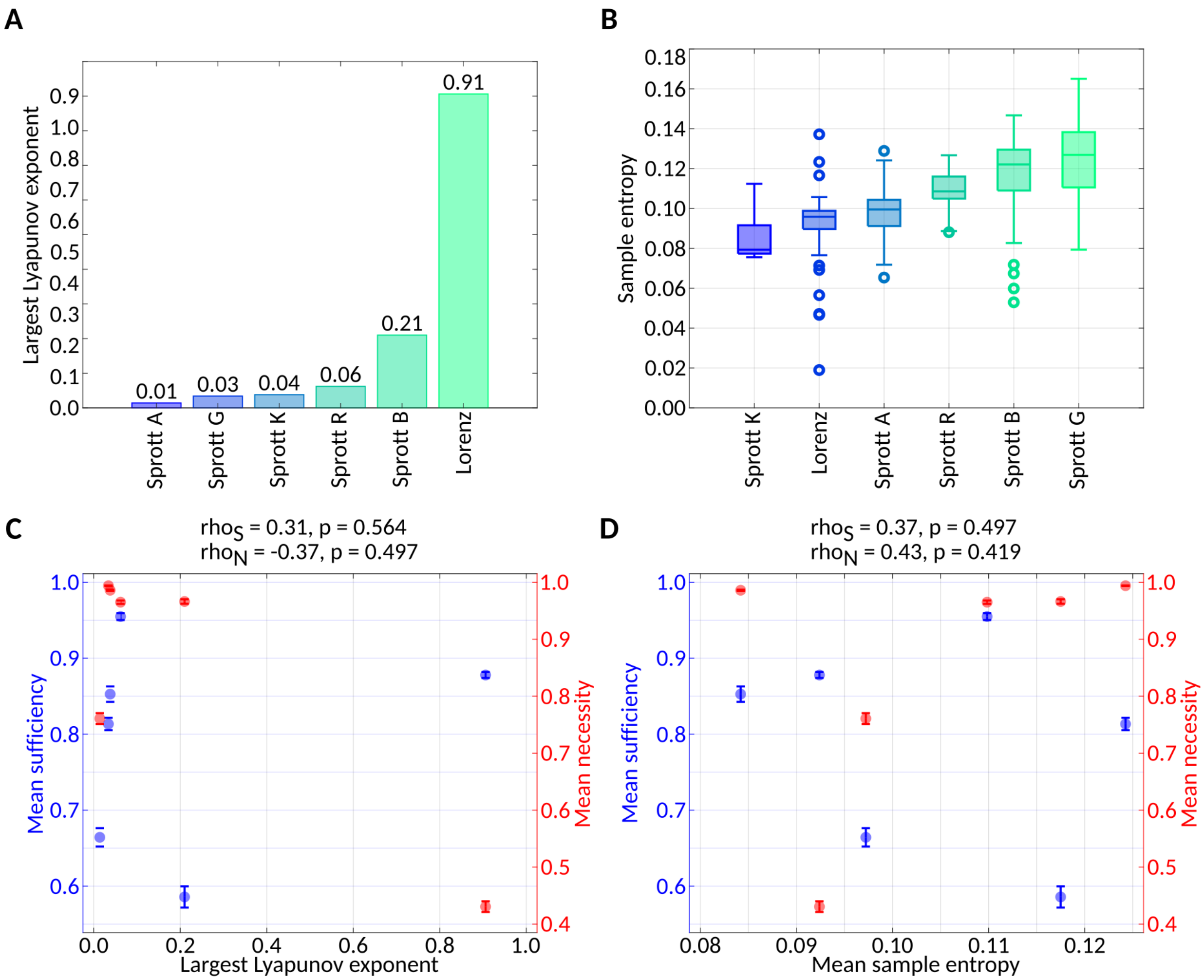

Figure S6. Exploratory analyses of task complexity across environments.

(**A**) Largest Lyapunov exponents (LLEs) for each task environment. (**B**) Distribution of sample entropy values computed from 100 randomly sampled test time series per environment (embedding dimension $m = 2$, tolerance $r = 0.2 \cdot \mathrm{std}$, sequence length 1500). (**C**) Relationship between LLE and sufficiency ($\mathrm{P}(S|E)$, blue) or necessity ($1 - \mathrm{P}(S|\neg E)$, red). Markers indicate environment means, with error bars showing the standard error across 1000 reservoirs with random hyperparameters, each evaluated on the 100 test time series from each environment. Titles report Spearman correlations (ρ, p) for both sufficiency (rho_S) and necessity (rho_N). (**D**) Same as (C), but with environment-mean sample entropy on the x-axis instead of LLE.

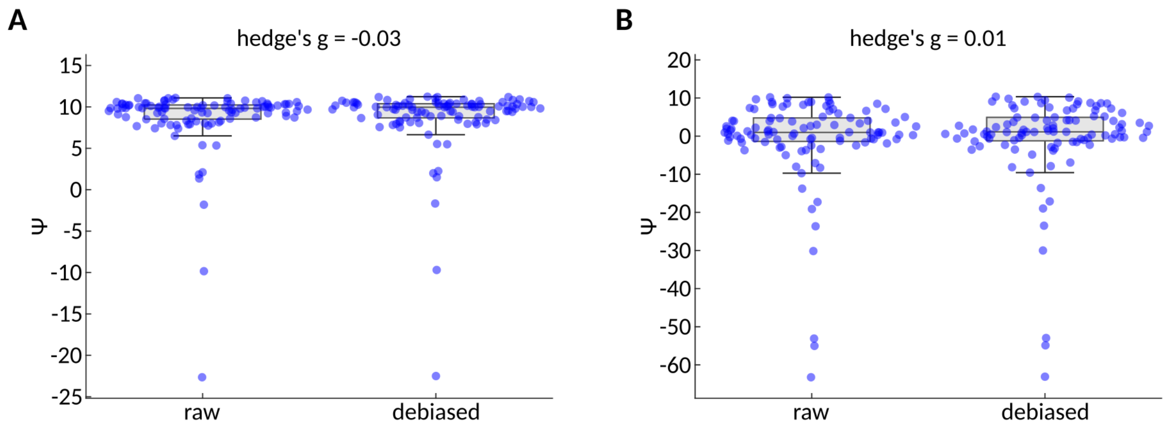

Figure S7. Bias analysis of ψ estimates in the Lorenz environment.

(**A**) Raw and debiased ψ values for all reservoirs of the loss-optimized population. (**B**) Same as (A), but for the ψ-optimized population. Debiasing was performed by repeatedly shuffling the time indices of each test time series (50 surrogates per series), computing ψ on the shuffled data, and subtracting the average shuffled value from the raw estimate. Each dot represents one reservoir in the population, with raw and debiased ψ values computed on the same 100 test time series used during evolutionary optimization (values shown are averages across the 100 series). A permutation-based paired t-test was applied to compare raw and debiased estimates. As expected, the test is always significant because $\psi_{\text{debiased}} = \psi_{\text{raw}} - c$ with $c > 0$, but the plot titles report hedge's g, showing that the effect sizes are very small.
